# Ultrasound Imaging of Plantar Fascia in Apparently Healthy Individuals, Diabetics and Patients With Plantar Fasciitis: A Case-Control Study

**DOI:** 10.7759/cureus.88609

**Published:** 2025-07-23

**Authors:** Soumya Chincholikar, Anil K Sakalecha, Guruyogendra Muthyal, Suprith Shankar, Sneha D N, Sri Madhurima Puttagunta, Richik Sarkar

**Affiliations:** 1 Radiodiagnosis, Sri Devaraj Urs Medical College, Kolar, IND; 2 Medicine, Sri Devaraj Urs Medical College, Kolar, IND; 3 Orthopaedics, Sri Devaraj Urs Medical College, Kolar, IND

**Keywords:** diabetes mellitus, doppler vascularity, healthy, neovascularization, plantar fascia

## Abstract

Introduction: The plantar fascia (PF) is a critical load-bearing structure of the foot, contributing to arch support and gait mechanics. Diabetes mellitus (DM) and plantar fasciitis (PFis) are two distinct conditions that affect the structure and function of the PF. While PFis is typically linked to mechanical overload and localized degeneration, diabetes may lead to more widespread changes in connective tissue consistency and thickness. Although both conditions impact PF morphology, no meta-analysis to date has systematically distinguished the ultrasound features of diabetic fascial thickening versus plantar fasciitis, highlighting a critical gap that this study addresses.

Methods: This case-control ultrasound study included 90 participants aged 30-60 years (30 per group): (1) healthy controls, (2) patients with type 2 DM (duration five or more years, no heel pain), and (3) non-diabetic individuals with clinically diagnosed unilateral PFis. High-resolution ultrasound was performed to assess PF thickness was measured 1 cm distal to the calcaneal insertion (a standardized site with good reproducibility), echogenicity (normal vs. reduced), and power Doppler vascularity. The examiner was blinded to group assignment. Group comparisons were conducted using ANOVA and chi-square tests.

Results: PF thickness was significantly increased in both diabetic (3.4 ± 0.6 mm) and PFis (5.6 ± 1.1 mm) groups compared to controls (2.6 ± 0.5 mm, p<0.001). Hypoechogenicity was observed in 90% of PFis subjects but only 7% of individuals with diabetes and none of the controls (p<0.001). Doppler vascularity was absent in controls and nearly all diabetics (3%) but present in 33% of PFis cases (p < 0.01), typically indicating neovascularization.

Conclusion: Ultrasound effectively distinguishes PF changes in diabetes and PFis. While both conditions show PF thickening, PFis is characterised by hypoechoic hypervascular fascia, reflecting localised degeneration. Diabetic PF changes are diffuse and subclinical, without significant echo-textural disruption or inflammation. These findings underscore the diagnostic utility of ultrasound in evaluating heel pain and in the early detection of diabetic foot tissue remodelling.

## Introduction

The plantar fascia (PF) is a strong fibrous aponeurosis located along the sole. Originating from the medial calcaneal tubercle and extending forward to the bases of the toes, it plays a crucial role in maintaining the longitudinal arch and enabling normal foot biomechanics. During gait, the PF functions as part of the windlass mechanism, providing dynamic support to the arch. Given its constant load-bearing function, the PF is particularly susceptible to repetitive strain, overload injuries, and degenerative changes [1,2].

Plantar fasciitis, more accurately referred to as plantar fasciopathy, is the most common cause of chronic heel pain. It results from repeated microtrauma at the proximal insertion of the PF, often without significant inflammation. Histopathologic studies have demonstrated that this condition is more degenerative than inflammatory, characterised by findings such as collagen disorganisation, fibroblast hypertrophy, and micro tears [3,4]. This has led to the increasing use of the term "plantar fasciitis" in the medical literature. Clinically, it presents with characteristic symptoms of plantar heel pain, particularly during the first few steps after waking or following prolonged periods of rest. Risk factors for plantar fasciitis include obesity, occupations requiring prolonged standing, excessive running, reduced ankle dorsiflexion, and altered foot mechanics that place increased tensile stress on the PF [5]. Systemic conditions, such as seronegative spondyloarthropathies, can also coexist and contribute to the development of plantar fasciitis [6]. While most cases respond to conservative treatment, chronic fasciopathy can significantly impair mobility and quality of life.

Diabetes mellitus (DM), a systemic metabolic disorder, is known to affect musculoskeletal structures through mechanisms such as non-enzymatic glycation of collagen and accumulation of advanced glycation end-products. These alterations can affect soft tissue elasticity, leading to conditions such as limited joint mobility, tendon thickening, and diabetic neuroarthropathy [7,8]. In the foot, diabetes can cause subclinical changes in connective tissues, including the PF. Several studies have reported that patients with long-standing type 2 diabetes mellitus (T2DM) often exhibit increased PF thickness compared to healthy individuals [9,10]. This thickening may contribute to abnormal plantar pressure distribution, impaired foot mechanics, and increased risk of complications such as foot ulcers and Charcot arthropathy [11]. Notably, Craig et al. observed that PF thickness on ultrasound correlated with glycemic control and the risk of long-term complications in adolescents with type 1 diabetes, supporting the concept of PF thickness as a potential biomarker for cumulative glycemic exposure [12].

Ultrasound is a well-established imaging modality for evaluating the PF due to its superficial anatomical location. High-frequency ultrasound can accurately delineate the PF's morphology, fibrillar echotexture, and thickness. In healthy individuals, the PF appears as a well-defined, hyperechoic fibrillar band measuring approximately 2-3 mm in thickness near the calcaneal origin [13]. In plantar fasciitis, ultrasound typically reveals increased PF thickness (often greater than 4 mm), loss of normal echogenicity, and disrupted fibre architecture [14]. Additional findings, such as perifascial fluid collections, calcaneal spurs, or enthesophytes, may also be present. Power Doppler ultrasound can detect localised vascularity, indicating neovascularisation, which is occasionally observed in chronic cases of plantar fasciitis. However, the literature suggests that only a subset of patients with fasciitis demonstrates a significant Doppler signal, and its presence may correlate with greater symptom severity or chronicity [15,16].

While individual studies have examined PF changes in plantar fasciitis and diabetes separately, few have directly compared these populations with healthy individuals using a uniform ultrasound protocol. This knowledge gap has diagnostic implications. For example, PF thickening in a diabetic patient might be misinterpreted as plantar fasciitis if echotextural and vascular features are not carefully evaluated. Moreover, understanding the nature and extent of diabetes-induced changes in the PF may offer insights into how metabolic disease alters soft tissue integrity and predisposes to mechanical complications in the foot.

Given this background, we designed a case-control study using high-resolution ultrasound to evaluate and compare PF characteristics in three well-defined cohorts: healthy individuals, asymptomatic patients with T2DM, and non-diabetic patients clinically diagnosed with plantar fasciitis. We hypothesised that patients with diabetes would exhibit measurable PF thickening and potentially early structural changes on ultrasound, intermediate between those of healthy controls and individuals with plantar fasciitis, reflecting the impact of chronic hyperglycemia on fascial tissue. The objectives of our study were to assess and compare PF thickness, echogenicity, and vascularity across the three groups and to discuss the clinical significance of these sonographic differences in terms of diagnosis, risk stratification, and understanding of foot biomechanics in diabetes. By characterising the ultrasound phenotype of the "diabetic fascia," this study aims to enhance diagnostic accuracy and contribute to the understanding of musculoskeletal alterations associated with diabetes.

## Materials and methods

This case-control observational study was conducted in the Department of Radiology at a tertiary care teaching hospital following approval from the Institutional Ethics Committee. Written informed consent was obtained from all participants prior to their enrollment. A total of 90 subjects were recruited and equally divided into three groups of 30 participants each: healthy controls, patients with T2DM without heel pain, and non-diabetic patients with clinically diagnosed plantar fasciitis. Eligible participants were adults between 30 and 60 years of age, to ensure a comparable middle-aged cohort and minimize confounding due to age-related connective tissue changes.

The healthy control group included individuals without a history of heel pain, systemic metabolic disorders, or prior foot pathology. These participants were primarily hospital staff and patient attendants, selected to match the age and sex distribution of the other groups as closely as possible. The diabetic group comprised patients diagnosed with T2DM for at least five years, treated with oral hypoglycemic agents or insulin, but without any history of plantar heel pain. Patients with foot ulcers, Charcot arthropathy, or any significant foot deformities were excluded from this group. The third group consisted of non-diabetic individuals clinically diagnosed with plantar fasciitis, presenting with typical features such as chronic inferior heel pain and point tenderness over the medial calcaneal tubercle. In participants with bilateral plantar fasciitis, only the foot with more severe symptoms - based on patient-reported pain intensity - was selected for ultrasound assessment to prevent within-subject duplication. For unilateral cases, the affected foot was examined.

Exclusion criteria applied across all groups included a history of foot or ankle surgery, recent foot trauma, inflammatory arthropathies (such as rheumatoid arthritis or spondyloarthropathies), severe peripheral arterial disease, and congenital or acquired foot deformities such as pes planus or pes cavus. The sample size was determined using power analysis based on preliminary data suggesting an area under the receiver operating characteristic (ROC) curve of 0.61 for PF thickness differences between diabetic and non-diabetic groups. With a two-tailed alpha level of 0.05 and a power of 80%, the total required sample size was calculated to be 90, divided equally among the three study groups.

All participants underwent standardized high-resolution ultrasound evaluation of the plantar fascia at the calcaneal insertion (using a 5-12 MHz linear-array transducer on a high-end ultrasound system). In unilateral cases, the symptomatic foot was examined. In bilateral cases, the more symptomatic foot was assessed for consistency. Foot dominance was not specifically controlled for but was assumed to have minimal effect given the focus on fascial thickness rather than functional asymmetry. Scans were performed with subjects lying prone, their feet extended beyond the examination table, and their ankles maintained in a neutral (90°) position. In the plantar fasciitis group, the symptomatic heel was scanned. For healthy controls and diabetic participants, one foot was randomly selected unless clinical asymmetry was evident. The ultrasound probe was positioned longitudinally along the medial heel to visualize the PF from its calcaneal origin. Care was taken to align the probe with the fascial fibers and minimize anisotropic artifacts, using ample coupling gel to avoid compressing the soft tissues.

Plantar fascia thickness was measured at a standardized location, 1 cm distal to its calcaneal insertion, to avoid the enthesis and fibrocartilaginous interface. The perpendicular distance between the superficial and deep margins of the fascia was measured using electronic calipers. Each measurement was taken twice by an experienced musculoskeletal radiologist, and the average value was recorded. Intra-observer variability was low, with a variation of less than 0.2 mm. Heel pad (subcalcaneal fat pad) thickness was also measured in all subjects as a secondary parameter.

Echogenicity of the PF was assessed qualitatively using longitudinal scans. A normal PF was defined as a uniformly hyperechoic, striated structure. Any reduction in echogenicity, loss of parallel fiber architecture, or heterogeneous appearance was recorded as “reduced echogenicity.” No participant exhibited findings suggestive of plantar fibroma or fascial tears. Following grayscale evaluation, power Doppler ultrasound was performed to assess vascularity within or around the PF. Settings were optimized for detecting low-velocity blood flow (pulse repetition frequency ~500 Hz, low wall filter, and high sensitivity). Any consistent color Doppler signal observed within the PF or its adjacent fascial tissue in at least two planes was recorded as “vascularity present.” All ultrasound scans were performed by a single radiologist with over ten years of experience in musculoskeletal imaging. The radiologist was blinded to the participants' group assignments to reduce observational bias. While complete blinding was not feasible in obviously symptomatic fasciitis cases, the radiologist was not informed of diabetic status in the control and diabetic groups. To assess inter-observer reliability, 10 randomly selected cases were independently re-evaluated by a second radiologist. The intraclass correlation coefficient (ICC) for PF thickness was 0.88, and Cohen’s kappa coefficient for echogenicity classification was 0.80, indicating excellent inter-observer agreement.

The primary outcomes of this study were PF thickness, measured in millimeters, and its echogenicity, categorized as normal or reduced. Secondary outcomes included Doppler vascularity and heel pad thickness. One-way analysis of variance (ANOVA) was used to compare mean PF thickness among the three groups, followed by Tukey's post hoc test for pairwise comparisons. For categorical variables such as echogenicity and Doppler vascularity, Chi-square or Fisher's exact tests were applied. Within the diabetic group, exploratory correlation analyses were conducted to evaluate associations between PF thickness and clinical parameters such as duration of diabetes, HbA1c levels, and the presence of peripheral neuropathy, using Pearson or Spearman correlation coefficients based on data distribution. A p-value of less than 0.05 was considered statistically significant. All statistical analyses were performed using SPSS Statistics version 25.0 (IBM Corp., Armonk, NY, USA).

## Results

The study enrolled 90 participants, evenly distributed among three study groups: healthy controls, individuals with diabetes but without heel pain, and non-diabetic patients with plantar fasciitis. There were no significant differences in baseline demographic characteristics across the groups. The mean age was 45.3 ± 8.0 years in the control group, 47.1 ± 6.5 years in the diabetic group, and 46.8 ± 7.2 years in the plantar fasciitis group (p = 0.60, ANOVA). Body mass index was comparable across the three groups (mean ~27 kg/m², p = 0.74). The mean HbA1c among diabetic subjects was 8.1% (range: 6.7-10.4), with a median diabetes duration of eight years. Clinical peripheral neuropathy was present in 12 of the 30 diabetic participants. Among those with plantar fasciitis, 20 had unilateral symptoms, and 10 had bilateral involvement; for bilateral cases, one foot was randomly selected for analysis. The mean symptom duration was five months, and 40% of patients reported a recent increase in activity or exercise preceding the onset of symptoms. No subject in the plantar fasciitis group had diabetes or inflammatory arthritis as depicted in Table 1.

**Table 1:** Baseline Participant Characteristics

Parameter	Healthy Controls (n=30)	Diabetics (n=30)	Plantar Fasciitis (n=30)	Test Used	Test Statistic	p-value
Mean Age (years)	45.3 ± 8.0	47.1 ± 6.5	46.8 ± 7.2	One-way ANOVA	F(2,87) = 0.73	0.48
Mean BMI (kg/m²)	~27	~27	~27	One-way ANOVA	F(2,87) = 0.12	0.88
HbA1c (%)	NA	8.1 (6.7–10.4)	NA	—	—	—
Diabetes Duration (years)	NA	8 (median)	NA	—	—	—
Peripheral Neuropathy (n)	0	12	0	Chi-square	χ²(2) = 26.4	<0.001
Bilateral Cases (n)	0	0	10	Chi-square	χ²(2) = 20.0	<0.001
Mean Symptom Duration (months)	NA	NA	5	—	—	—
Recent Activity Increase (%)	0%	0%	40%	Chi-square	χ²(2) = 29.1	<0.001

PF thickness demonstrated a statistically significant increase from controls to individuals with diabetes to those with fasciitis. In healthy controls, the mean PF thickness was 2.56 ± 0.48 mm (range: 1.9-3.4 mm), consistent with previously published normative data. Diabetic subjects exhibited a higher mean thickness of 3.36 ± 0.59 mm (range: 2.3-4.5 mm), approximately 30% greater than that of controls (p < 0.0001). Notably, 77% of diabetic patients had a PF thickness exceeding 3.5 mm (i.e., the mean + 2 SD of the control group), indicating a clear trend of diffuse thickening. The plantar fasciitis group demonstrated the most significant increase in PF thickness, with a mean value of 5.58 ± 1.05 mm (range: 4.2-7.1 mm), significantly higher than both controls and diabetics (p < 0.0001 for both comparisons). ANOVA confirmed a strong overall group difference (F = 128, p < 0.0001), and post-hoc Tukey's tests revealed that each pairwise comparison was statistically significant. Among people with diabetes, PF thickness showed a modest, non-significant correlation with HbA1c (r = 0.32, p = 0.08) and diabetes duration (r = 0.28, p = 0.12). Additionally, those with neuropathy had slightly higher mean thickness (3.5 mm vs. 3.2 mm), although the difference did not reach statistical significance (p = 0.20) as illustrated in Table 2.

**Table 2:** Plantar Fascia Thickness Across Study Groups Statistical Tests Used: One-way ANOVA: F = 128, p < 0.0001 Post-hoc Tukey’s test: All intergroup comparisons significant (p < 0.001)

Group	Mean PF Thickness (mm) ± SD	Range (mm)	% with PF Thickness > 3.5 mm	Test Used	Test Statistic	p-value
Healthy Controls (n=30)	2.56 ± 0.48	1.9 – 3.4	0%	ANOVA + Tukey	F(2,87) = 89.2	<0.0001
Diabetics (n=30)	3.36 ± 0.59	2.3 – 4.5	77%			<0.0001
Plantar Fasciitis (n=30)	5.58 ± 1.05	4.2 – 7.1	93% > 4.0 mm			<0.0001

Evaluation of PF echogenicity revealed consistent hyperechoic fibrillar patterns in all healthy controls. In people with diabetes, 93% retained normal echogenicity despite increased PF thickness. Two diabetic patients (6.7%) displayed mildly hypoechoic and heterogeneous PF-both with poor glycemic control and long-standing diabetes-possibly representing early subclinical diabetic fasciopathy. In contrast, patients with plantar fasciitis showed a high prevalence of abnormal echotexture. Specifically, 27 of 30 (90%) had hypoechoic, swollen fascia with loss of the standard fibrillar architecture, while the remaining three showed either mixed or patchy echogenicity. The incidence of reduced echogenicity was significantly higher in the plantar fasciitis group than in both the diabetic and control groups (Chi-square p < 0.0001). There was no statistically significant difference in echogenicity between diabetics and healthy controls (p = 0.15). These findings suggest that while diabetes may contribute to PF thickening, it typically does not result in the collagen disorganisation and oedema characteristic of plantar fasciitis as described in Table 3.

**Table 3:** Plantar Fascia Echogenicity Pattern in Study Groups Statistical Tests Used: Chi-square test across groups: p < 0.0001 Diabetics vs. Controls: p = 0.15 (not significant) PFis: plantar fasciitis

Group	Total Subjects	Normal Echogenicity (n, %)	Reduced Echogenicity (n, %)	Test Used	p-value (vs. PFis)
Healthy Controls	30	30 (100%)	0 (0%)	Chi-square	< 0.0001
Diabetics (Asymptomatic)	30	28 (93.3%)	2 (6.7%)	Chi-square	< 0.0001
Plantar Fasciitis	30	3 (10%)	27 (90%)	–	–

Colour/power Doppler ultrasound showed no vascular flow in the PF of any healthy control, as expected. Similarly, 29 of 30 diabetic subjects had no detectable Doppler signal within or around the fascia. One diabetic patient exhibited a minimal colour spot at the fascia insertion, but this was considered incidental or artifactual. In contrast, Doppler findings were positive in 10 of 30 (33%) patients with plantar fasciitis. These patients demonstrated low-to-moderate perifascial hyperemia, with mild (grade 1) vascular signal in most cases and moderate (grade 2) signal in two cases. No severe or pulsatile flow was observed. Doppler-positive cases tended to have chronic symptoms. A statistical comparison using Fisher's exact test revealed significantly more vascularity in the plantar fasciitis group than in either the control or diabetic groups (p < 0.01). Nevertheless, the absence of Doppler flow in the remaining two-thirds of fasciitis cases confirms that Doppler is not a necessary finding and may be more indicative of active inflammation or neovascularization in chronic fasciitis as shown in Table 4.

**Table 4:** Comparison of Doppler Vascularity and Heel Pad Thickness Across Study Groups

Parameter	Healthy Controls (n=30)	Diabetics (n=30)	Plantar Fasciitis (n=30)	Test Used	Test Statistic	p-value
Positive Doppler Signal	0 (0%)	1 (3.3%)	10 (33.3%)	Chi-square	χ²(2) = 15.2	<0.01
Heel Pad Thickness (mm)	13.4 ± 2.1	13.1 ± 1.8	12.9 ± 2.5	One-way ANOVA	F(2,87) = 0.60	0.55
Calcaneal Spurs Detected	0	1	2	—	—	—
Plantar Fibromas/Tears	0	0	0	—	—	—

An ancillary analysis of heel pad thickness revealed no significant differences across groups. Mean subcalcaneal fat pad thickness was 13.4 ± 2.1 mm in controls, 13.1 ± 1.8 mm in diabetics, and 12.9 ± 2.5 mm in plantar fasciitis patients (p = 0.55), indicating that neither diabetes nor plantar fasciitis had a notable impact on heel pad thickness in this cohort. No plantar fibromas or fascia tears were identified in any subject. Two patients in the plantar fasciitis group had small calcaneal spurs visible on ultrasound, and one diabetic subject had a minor enthesophyte at the PF insertion. These findings were incidental and not felt to influence the primary outcomes.

## Discussion

This study presents a comprehensive ultrasound-based evaluation of the PF in three distinct cohorts: healthy individuals, patients with T2DM, and non-diabetic patients with clinically diagnosed plantar fasciitis. The findings support our hypothesis that while both diabetes and plantar fasciitis result in increased PF thickness, they differ significantly in qualitative ultrasound features such as echogenicity and vascularity. To our knowledge, this is among the few studies to include an asymptomatic diabetic group in parallel with plantar fasciitis patients and healthy controls, thus offering a nuanced perspective on diabetes-related fascial changes.

Our results show that both diabetic individuals and patients with plantar fasciitis had significantly thicker plantar fasciae compared to healthy controls, reinforcing PF thickening as a common hallmark of both conditions. Patients with plantar fasciitis exhibited the greatest mean thickness (~5.6 mm), consistent with prior studies that validate PF thickness as a reliable diagnostic marker for fasciitis. For example, a threshold of 4 mm at the calcaneal origin has been associated with high sensitivity and specificity for diagnosing plantar fasciitis [17]. In our cohort, 93% of fasciitis patients had PF thickness exceeding this cutoff, in agreement with earlier findings reporting symptomatic fasciae in the range of 4.5-5 mm [18-20].

In the diabetic group, PF thickness was also significantly elevated (mean ~3.4 mm) compared to healthy controls (mean ~2.6 mm), consistent with findings by Giacomozzi et al. and Duffin et al., who reported increased plantar soft tissue thickness in diabetic individuals [21,22]. Our results are also in line with Abate et al., who observed PF thickening in newly diagnosed type 2 diabetics, where PF changes correlated more strongly with BMI than with Achilles tendon thickness [23]. Notably, as our study groups were BMI-matched, the observed thickening in diabetics can be attributed more confidently to the metabolic effects of diabetes itself. The pathophysiological basis for this fascial thickening in diabetes likely includes non-enzymatic glycation of collagen, extracellular matrix expansion due to fluid retention, and low-grade chronic inflammation [24]. These structural alterations may compromise fascial elasticity and increase susceptibility to subclinical microtrauma, even in the absence of symptoms [25].

A key differentiator between the diabetic and fasciitis groups was fascial echotexture. In diabetics, the PF, despite being thickened, largely preserved its normal hyperechoic, fibrillar architecture. Only 6.7% of diabetic participants demonstrated hypoechoic changes, all of whom had long-standing, poorly controlled diabetes. This suggests that diabetes may lead to fascial hypertrophy without disrupting collagen alignment. Collagen cross-linking via advanced glycation end-products (AGEs), a hallmark of chronic hyperglycemia, stiffens soft tissues without necessarily altering echogenicity [26]. Recent elastography studies indicate that despite preserved echotexture, diabetic PF may demonstrate altered stiffness profiles, likely reflecting changes in the mechanical properties of the collagen matrix. In contrast, plantar fasciitis was marked by marked hypo-echogenicity in 90% of patients. These hypoechoic, thickened, and disorganized fasciae reflect underlying histopathologic features such as collagen degeneration, edema, and microtears. Several studies have quantitatively confirmed reduced echogenicity in fasciitis patients compared to controls, extending beyond just the enthesis. Our data reinforce that hypo-echogenicity is a more specific marker of active fascial pathology than mere thickening and is seldom seen in asymptomatic diabetic individuals.

Power Doppler ultrasound further highlighted this distinction. None of the healthy controls and only one diabetic subject showed vascularity, while 33% of plantar fasciitis patients demonstrated perifascial hyperemia. It is important to recognize that Doppler signal detection can be influenced by certain confounding factors such as ultrasound equipment sensitivity, machine gain settings, operator technique, and recent patient physical activity, which might transiently increase local blood flow. In this study, we minimized these effects by using a single high-resolution ultrasound system operated by a musculoskeletal radiologist with over 10 years of experience, applying standardized machine settings, and advising patients to avoid strenuous foot activity before the scan. This vascular signal likely represents neovascularization associated with chronic inflammation and tissue repair. Walther et al. reported Doppler positivity in ~40% of patients with chronic fasciitis, interpreting it as a sign of localized inflammation and reparative angiogenesis [27]. Our findings are consistent with this observation and support the potential utility of Doppler imaging in assessing fasciitis chronicity. Moreover, Doppler-positive patients in our study typically had longer symptom duration. Previous interventional studies have shown that targeting these hyperemic regions with corticosteroids or platelet-rich plasma (PRP) injections may result in symptom relief and restoration of normal sonographic architecture [28]. Thus, Doppler-guided interventions may hold therapeutic value.

From a biomechanical perspective, PF thickening-regardless of its origin-may alter foot mechanics. As a crucial component of the longitudinal arch, the PF functions in shock absorption and propulsion. In diabetes, increased stiffness and reduced elasticity of the PF have been linked to higher plantar pressures and limited ankle dorsiflexion. D’Ambrogi et al. demonstrated that altered PF biomechanics contribute to abnormal plantar pressure distribution, potentially increasing the risk of foot ulceration [29]. Our findings further support this mechanism by showing that even asymptomatic diabetics exhibit structural PF changes.

We also observed a modest positive correlation between PF thickness and HbA1c levels, supporting previous observations that PF thickness may reflect cumulative glycemic exposure. Craig et al. proposed that PF thickness could serve as a surrogate marker for systemic glycation burden, and found a relationship between PF thickness and the development of diabetic microvascular complications [12]. Benitez-Aguirre et al. confirmed this association in longitudinal studies involving adolescents with diabetes, suggesting a potential role for PF thickness as a metabolic biomarker [30].

Ultrasound evaluation of the PF not only aids in diagnosis but may also offer prognostic insight in plantar fasciitis. Studies have demonstrated that hypoechoic and markedly thick fasciae may take longer to respond to therapy and may benefit from biologic interventions or physical therapies [31]. Aggarwal et al. reported that corticosteroid injections led to reductions in PF thickness and normalization of echogenicity in most patients [32]. In this context, serial ultrasound assessments could help monitor disease progression and therapeutic response, thereby guiding individualized management.

Despite these strengths, our study has several limitations. First, as a case-control design, it cannot establish causality or predict whether asymptomatic PF thickening in diabetes will progress to symptomatic fasciitis. Second, although the sample size was sufficient for primary comparisons, larger cohorts-especially for the diabetic and plantar fasciitis groups-would have improved statistical power and allowed more robust subgroup analyses. Third, we focused on the proximal 1 cm of the PF, which is the most reproducible and clinically relevant site for measurement; however, pathological changes can extend more distally and were not evaluated here. Additionally, potential confounders such as ethnicity, type of occupation, and daily physical activity level were not controlled for, although these factors may influence PF thickness, heel pad characteristics, and Doppler findings. Furthermore, while echogenicity was qualitatively assessed by an experienced radiologist, quantitative methods such as greyscale histogram analysis could provide more objective insights in future studies. Finally, as the study was conducted at a single center with a predominantly South Indian population, the generalizability to other ethnic or geographic populations may be limited.

## Conclusions

High-resolution ultrasonography effectively differentiates the morphological and vascular changes of the plantar fascia in healthy individuals, asymptomatic patients with T2DM, and patients with plantar fasciitis. While plantar fasciitis is characterized by significant thickening, loss of fibrillar architecture, and increased Doppler vascularity, diabetic patients show moderate thickening with preserved echogenicity and minimal vascularity, highlighting distinct underlying pathophysiological processes. These findings support the role of PF ultrasound as a valuable, non-invasive diagnostic tool for confirming plantar fasciitis and potentially monitoring diabetes-related fascial changes. Future longitudinal research is needed to clarify whether early PF alterations in diabetes predict foot complications and whether timely interventions can modify these changes.
